# Trends in Fat Modifications Enabling Alternative Partially Hydrogenated Fat Products Proposed for Advanced Application

**DOI:** 10.3390/gels9060453

**Published:** 2023-06-01

**Authors:** Anna Zbikowska, Sylwia Onacik-Gür, Małgorzata Kowalska, Katarzyna Zbikowska, Melánia Feszterová

**Affiliations:** 1 Institute of Food Sciences, Faculty of Food Assessment and Technology, Warsaw University of Life Sciences (WULS-SGGW), Nowoursynowska St. 159c, 02-776 Warsaw, Poland; 2 Department of Meat and Fat Technology, Prof. Waclaw Dąbrowski Institute of Agricultural and Food Biotechnology-State Research Institute, 36 Rakowiecka St., 02-532 Warsaw, Poland; sylwia.onacik-gur@ibprs.pl; 3 Faculty of Chemical Engineering and Commodity Science, Kazimierz Pulaski University of Technology and Humanities, Chrobrego St. 27, 26-600 Radom, Poland; 4 Faculty of Medicine, Medical University of Warsaw, Zwirki i Wigury St. 61, 02-091 Warsaw, Poland; kasiazbikus@wp.pl; 5 Department of Chemistry, Faculty of Natural Sciences and Informatics, Constantine the Philosopher University in Nitra, 94901 Nitra, Slovakia; mfeszterova@ukf.sk

**Keywords:** structured fats, solid fat replacers, oleogels, enzymatic interesterification

## Abstract

The natural properties of oils and fats do not always allow for their direct use in industry (e.g., for food, cosmetics, and pharmaceuticals). Furthermore, such raw materials are often too expensive. Nowadays, the requirements for the quality and safety of fat products are increasing. For this reason, oils and fats are subjected to various modifications that make it possible to obtain a product with the desired characteristics and good quality that meets the needs of product buyers and technologists. The modification techniques of oils and fats change their physical (e.g., raise the melting point) and chemical properties (e.g., fatty acid composition). Conventional fat modification methods (hydrogenation, fractionation, and chemical interesterification) do not always meet the expectations of consumers, nutritionists, and technologists. In particular, Hydrogenation, while it allows us to obtain delicious products from the point of view of technology, is criticised for nutritional reasons. During the partial hydrogenation process, trans-isomers (TFA), dangerous for health, are formed. One of the modifications that meets current environmental requirements and trends in product safety and sustainable production is the enzymatic interesterification of fats. The unquestionable advantages of this process are the wide spectrum of possibilities for designing the product and its functional properties. After the interesterification process, the biologically active fatty acids in the fatty raw materials remain intact. However, this method is associated with high production costs. Oleogelation is a novel method of structuring liquid oils with small oil-gelling substances (even 1%). Based on the type of oleogelator, the methods of preparation can differ. Most oleogels of low molecular weight (waxes, monoglycerides, and sterols) and ethyl cellulose are prepared by dispersion in heated oil, while oleogels of high molecular weight require dehydration of the emulsion system or solvent exchange. This technique does not change the chemical composition of the oils, which allows them to keep their nutritional value. The properties of oleogels can be designed according to technological needs. Therefore, oleogelation is a future-proof solution that can reduce the consumption of TFA and saturated fatty acids while enriching the diet with unsaturated fatty acids. Oleogels can be named “fats of the future” as a new and healthy alternative for partially hydrogenated fats in foods.

## 1. Introduction

Even Hippocrates, called the father of medicine, saw the relationship between food and medicine [1]. Regardless of their origin or degree of processing, food products consist of various chemical compounds that affect human health. For example, fat plays an important role. Fats are essential food ingredients but also used in the production of cosmetics and medical products. Fat in the human diet is an appreciable energy source that may provide essential fatty acids and carriers of bioactive compounds, vitamins, and precursors. Furthermore, fat improves the palatability of food, texture, and aroma, making it easier to swallow a bite. Moreover, fats provide satiety and transfer heat in processes such as frying, preventing sticking [2,3,4]. 

Fats and oils are mostly comprised of triacylglycerides (TAG) made from the three fatty acids (FAs) in the glycerol backbone. The features of lipids are dependent upon the type and structural conformations of fatty acids [5]. These include saturated fatty acids (SFAs) and trans isomers (TFAs), which are criticised for their negative impact on health and should be removed as a source of food. The consumption of these fatty acids may cause obesity and inflammation and contribute to the risk of cardiovascular disease [5,6,7,8,9]. Dietary guidelines recommend a limited consumption of products with a high content of SFAs, regardless of origin. The total intake should be less than 10% of total energy per day [7,8]. The United States Food and Drug Administration (US-FDA) issued guidelines for removing trans fatty acids in 2015 [6]. Regulations on limiting the content of TFAs in food are also in force in the European Union [10]. It is recommended to replace SFAs and TFAs with unsaturated fatty acids [4,11]. 

Except for oleic acid, which is the most popular monoenoic fatty acid in vegetable oils, polyunsaturated FAs are recommended nutrients in the diet, particularly from the n-3 group [12]. C18:1cis reduces the risk of cardiovascular disease (including heart attack) by lowering blood pressure and total cholesterol [2,13,14,15]. 

The industry widely uses solid and semisolid fats, mainly because of the high content of SFA or TFA and the lower content of unsaturated fatty acids [4,16]. The most commonly used among these are naturally solid or industrially modified fats [17]. Palm oil, widely used in the industry, is a natural solid fat, but its use does not solve the problem of SFA, although it brings economic and technological benefits [18]. By the industrial modification of fats, products with desired functional properties can be obtained, e.g., in terms of melting point, solid phase content, and oxidative stability. The traditional techniques for obtaining modified fats include hydrogenation, fractionation, and chemical interesterification [3]. 

Methods used for transforming oils into solid and semisolid fats lead to the development of raw materials to produce a variety of foods. Using these fats in foods makes consumers aware of the relationship between diet and health, and some aspects cause concern. The first is associated with a high SFA content, and the second concerns the possible occurrence of TFA [19]. A relatively less common method of lipid structuring is enzymatic interesterification. Furthermore, oleogelation is a new modification method that allows for a compromise between the utility of fats and their nutritional value. As a result, it is possible to obtain a solid/semisolid fat with a high content of unsaturated fatty acids without SFA and TFA [4,19,20].

The most controversial method of oil modification is the partial hydrogenation of vegetable oils due to the formation of large amounts of TFA and SFA. Such fats are characterised by improved oxidative stability and very good plasticity. This process is technologically advantageous but carries the risks associated with TFA and its negative impact on human health [21].

Due to the need to increase the health value of fats and replace partially hydrogenated fats with other structured lipids, the present study reviews various methods of fat modification, with particular emphasis on oleogelation.

## 2. Conventional Methods of Fat Modification

### 2.1. Hydrogenation

**Figure 1:**
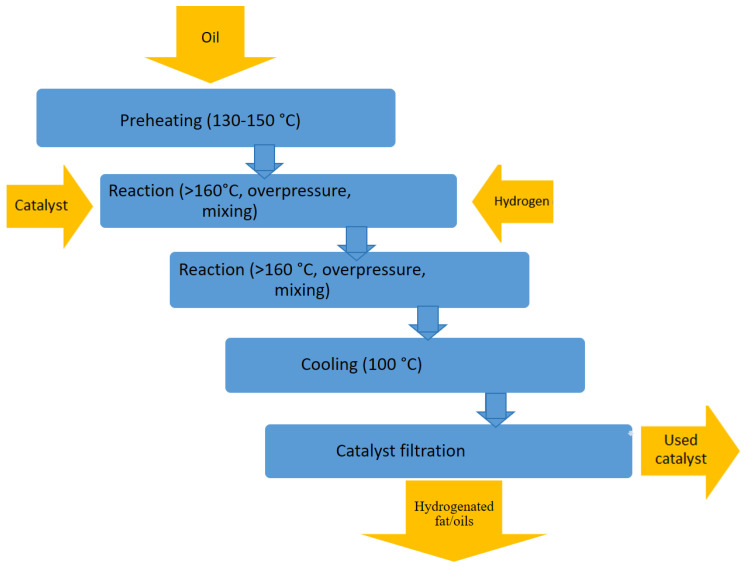
Diagram of the Modification process of fats and oil—hydrogenation.

Hydrogenation (HG) is a process aimed at changing the functional properties of liquid lipids. There are three types of HG, i.e., full HG, partial HG or partial HG without hardening [22]. HG leads to the production of solid or semisolid (plastic) fats from the oil. In the HG process, unsaturated FAs are converted into saturated fatty acids and TFAs. It is also possible to obtain oil with increased oxidative stability. An example of a hydrogenation process is shown in Figure 1. The high moisture of the oil has an adverse impact on the process. It may cause hydrolysis, which, in effect, forms free fatty acids. Under hydrogenation conditions (i.e., with high temperatures and a nickel catalyst), these compounds are saponified, which is why oils are dried before or after insertion in an autoclave. At the beginning of the HG process, oil is heated to 140 °C, the starting temperature of the HG reaction. The desired reaction can proceed at different temperatures, depending on the oil type and inhibition level of trans fatty acid formation [3,23,24].

Hydrogenation has a long history that extends back to the early 20th century. In 1901 Wilhelm Normann successfully hydrogenated fat for the first time, patenting his discovery in 1903. This technique has also been used in the fat and soap industry [25].

Hydrogenated fats have higher melting points than others, thus providing a favourable texture. Therefore, partially hydrogenated fat is suitable for many food products, e.g., margarine, shortenings, confectionery fats, frying fats and frying foods, sweets, doughnuts, and cakes [26].

**Figure 2:**
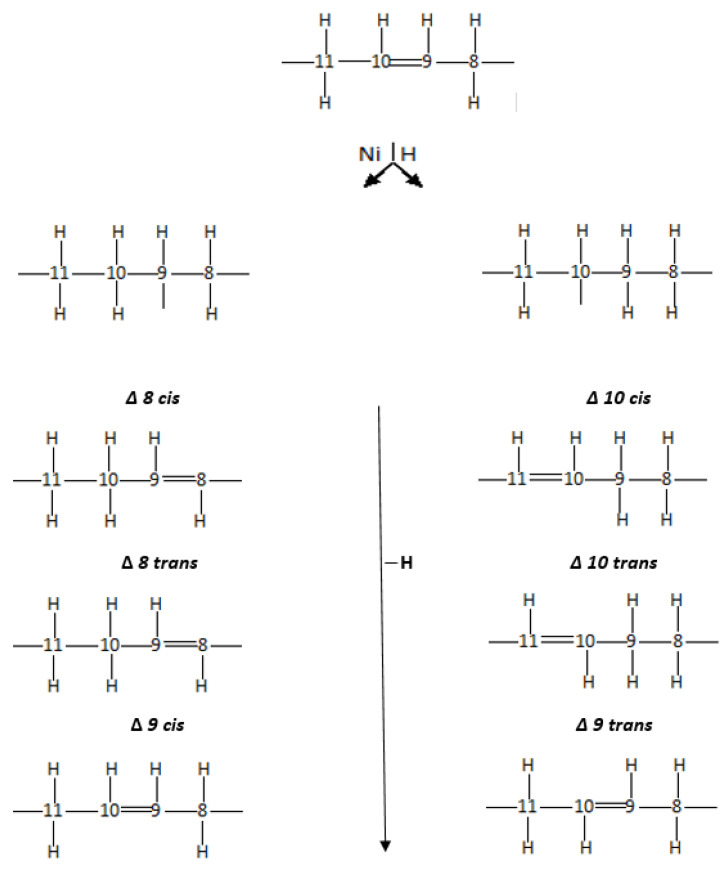
The mechanisms of the formation of trans isomers during the hydrogenation process.

Large amounts of TFAs are produced in the partial hydrogenation process. The mechanism of their formation, e.g., for oleic acid, is shown in Figure 2. From one cis isomer (oleic acids—C18:1c), several different geometric isomers are formed, chiefly elaidic acid (C18:1t). The TFA content in partially hydrogenated oil depends on the process parameters, i.e., temperature, hydrogen pressure, time, catalyst type, and concentration. The process of total hydrogenation leads to the complete saturation of unsaturated bonds and the formation of SFA, and oleic acids are transformed into stearic acid [3,25,27].

The unsaturation of the oil undergoing HG, as well as process conditions, affect the amount of formed TFAs (Table 1). Since hydrogen plays an important role in the trans isomerisation process, supplying the proper amount to the catalyst is important. This supply can be increased through higher pressure or better mixing conditions. Moreover, oils rich in unsaturated fatty acids demand higher amounts of hydrogen than highly saturated fats.

By increasing the amount of hydrogen (on the catalyst surface) during cis/trans isomerisation, the semi-hydrogenated compound-intermediate (Figure 2) reacts with the second hydrogen atommore quickly, and TFAs are formed [25,27].

It should be noted, however, that the health value of lipids is reduced due to chemical transformations taking place in triacylglycerol molecules (saturation of FAs unsaturated bonds and geometric cis-trans isomerisation). Unsaturated FAs lose their biological activity [3].

In the HG process, it is possible to obtain products with very diverse physicochemical characteristics and a high technological value. However, the low nutritional value and the increase in the content of harmful TFA in partially hydrogenated fats made it necessary to replace the partial hydrogenation process with other modification methods [3,4,5,14].

### 2.2. Fractionation

Fractionation is a thermomechanical separation process in which a multi-component mixture is separated into fractions with different physicochemical properties [25].

Fractionation is undoubtedly the oldest fat modification process and was the foundation of the oil and fat processing industry [28]. In most literature, Hippolyte Mège-Mouriès is credited with the invention of “a patented method to produce certain fats of animal origin”. He concocted the production of a kind of margarine by separating a liquid fraction from the tallow after cooling [28,29].

In the early stages of the development of fractionation technology, the olein and stearin fractions had to be separated by settling, using only the force of gravity [30]. However, the continuous development of separation techniques, from vacuum filtration to centrifuges and membrane press filters, has made fractionation a popular modification technique to obtain solid and semisolid fats [28].

**Figure 3:**
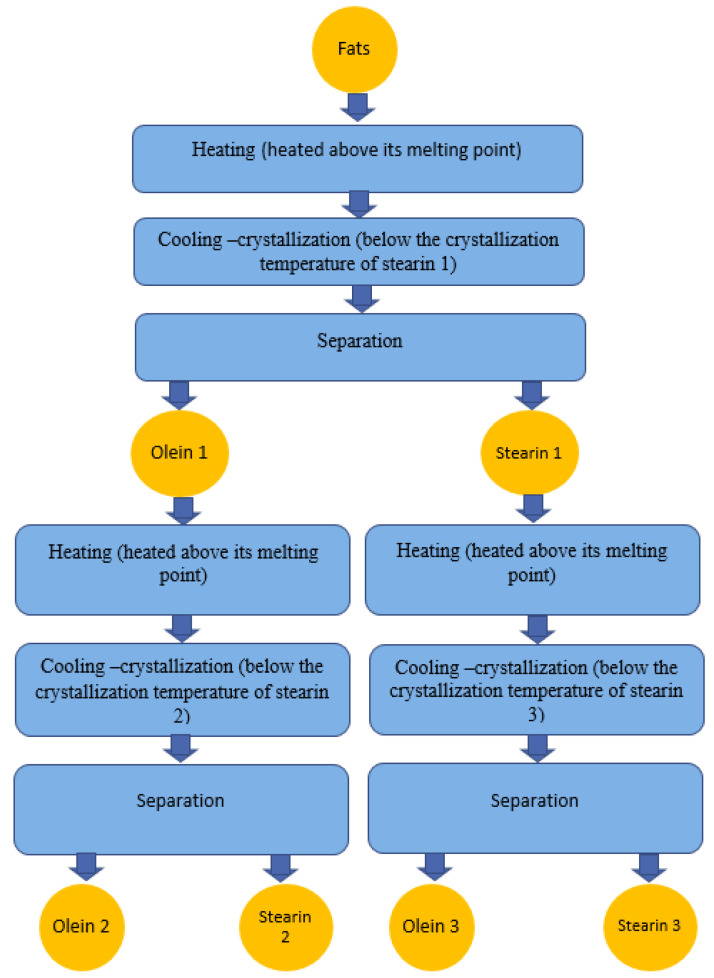
Diagram of the modification process of fats and oils: example of fractionation.

Fractionation is usually conducted in one or two steps. An example of such a modification process is shown in Figure 3. For typical standard applications, only stearin 1 and olein 1 are separated. Then, in the second step, one of these fractions (rarely both) is fractionated [25]. The process consists of the controlled cooling of the oil, thereby inducing a partial, or ‘fractional’, crystallisation. Finally, the remaining liquid faze (olein) is separated from the solid fraction (stearin) by means of filtration or centrifugation [28]. Some techniques relying on the use of detergents are applied for a particular production; however, only two main fractionation technologies are used in the edible oil industry [31]. These are:

- Dry fractionation (named crystallisation from the melt) is fractional crystallisation in this simple form. It is an economic and ecological technique [28,32]. This modification is a cheaper process than hydrogenation or interesterification [25,31]. The principle of dry fractionation is simple. The fat to be fractionated is heated above its melting point. Then it is cooled to the separation temperature, and the fractions are separated from each other. The cooling rate depends on the characteristics of the end products. The crystal mass (stearin) suspended in the oil is separated, which must be performed quickly to avoid partial remelting of the crystals. In large fractionation lines, crystal nuclei are formed in a precooling step in a big vessel that feeds several small vessels (crystallisation vessels) where the crystals are allowed to grow. Thus, one achieves higher efficiency by separating the sensitive step of nucleus formation from the time-consuming step of crystal growth [33,34].
- Solvent fractionation involves using an organic solvent (hexane, acetone, or isopropyl) to let the high-melting molecules crystallise in a low viscosity solvent. This method gives much purer solid fractions than can be obtained with vacuum filtration. However, it is a more expensive process and is thus less common than dry fractionation, and only comes into the picture when the added value of the resulting fractions makes up for the high cost [33,34].

### 2.3. Interesterification

**Figure 4:**
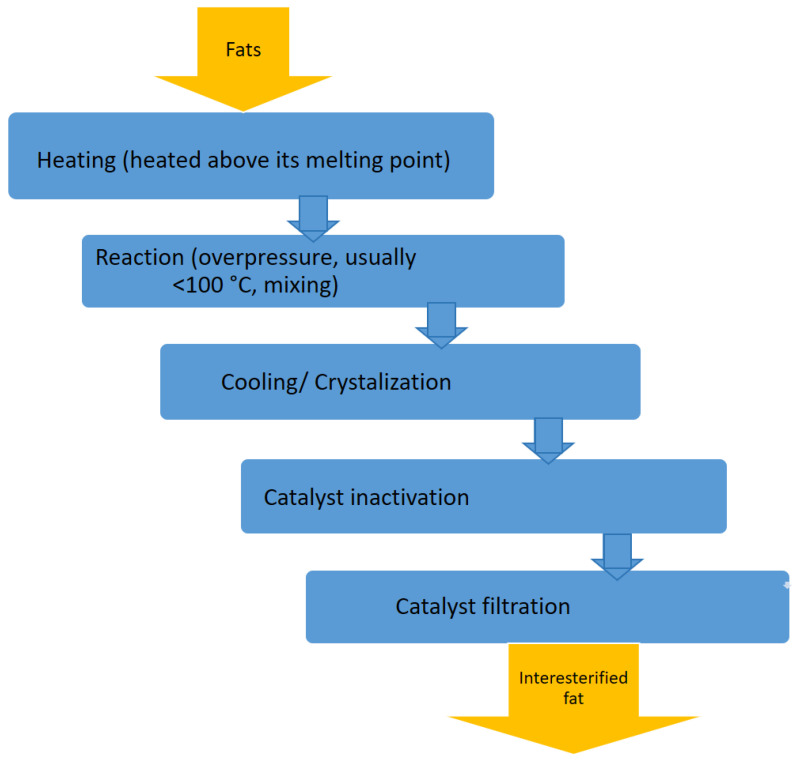
Diagram of Fat and Oil Modification Process—Interesterification.

Interesterification is a process in which the composition of the acids does not change, except for their arrangement in the triacylglycerol molecules. This process can ensure uniform properties of finished fats even if the composition of the raw material is subject to wide variations [25,35]. Interesterification can be conducted in a batch or continuous mode. In a batch process, the raw blend (oil/fats) is heated to 120–150 °C under a vacuum to remove moisture. After the drying step, the blend is cooled to 70–100 °C. The interesterification reaction then proceeds for 30–60 min. After the reaction, the catalyst is neutralised, and the filtration process product is ready to be bleached and deodorised [36]. A diagram of an interesterification is shown in Figure 4.

The history of interesterification started in the 1920s because of a shortage of solid and semisolid fats needed mainly for making margarine. The simple blending of oils with hard fats was unsuccessful because the mixtures were not homogenous. In contrast to simple blending, the interesterification process allowed the creation of homogeneous mixtures of TAG by incorporating the SFA of the fats into the oil TAG and vice versa [25].

Interesterification changes many parameters, such as the melting point and solid fat content and the crystallisation properties of fats/oils. For oils, the melting point is increased, and that of solid fats is decreased [25,37]. Interesterification can be random or directed. The former occurs when the reaction is carried out only in a liquid phase. However, when higher-melting TAGs are allowed to crystallise during the reaction, their main SFA is withdrawn from the liquid phase; consequently, the distribution of the FAs in the liquid phase will undergo no random-directed interesterification processes [38].

This process may so heavily change the properties of a fat/oil that the final product no longer has the starting material. What is important is that the interesterification does not result in the production of either TFA or positional isomers of FAs [24].

Nevertheless, the esterification method can be used to produce biodiesel, the new trend in biodiesel production from animal fat waste [39,40].

## 3. Trends in Fat Modification Technology

### 3.1. Enzymic Interesterification

This is a promising fat modification technique because it can obtain pro-ducts that cannot be produced with other methods.

Applications of fats can be restricted due to their natural triacylglycerol structures, which cannot provide desirable properties to the given products. Therefore, the fat products’ physicochemical and nutritional properties concern fatty acid profile and triacylgly-cerol structures.

One fat modification that meets current environmental requirements and fits in with safety trends for fat product modifications, along with the drive for sustainable production, is the enzymatic interesterification of fats. The unquestionable advantages of this process are the wide range of possibilities for product design and its functional properties [41]. Numerous scientific papers [42,43,44,45] suggest a great deal of interest in this direction in recent years. On 24 April 2019, the Commission Regulation (EU) 2019/649 was published regarding trans isomers of fatty acids other than the ones naturally occurring in fats of animal origin. The regulation became effective on 2 April 2021, and many food operators ended up reformulating the composition of manufactured products or completely withdrawing from the market. According to the regulation, the content of trans isomers of fatty acids other than ones naturally occurring in fat of animal origin must not exceed 2 grammes per 100 grammes of fat. In the United States, the Food and Drug Administration (FDA) has ruled that Partially Hydrogenated Oils (PHOs), a source of trans isomers, are no longer “generally recognised as safe” (GRAS) and cannot be included in food products [46]. In turn, the World Health Organization (WHO) on 14 May 2018, announced a six-step comprehensive plan called REPLACE, aimed at eliminating all partially hydrogenated fats from the world’s food supply by 2023 and replacing them with beneficial vegetable oils, excluding tropical oils, i.e., palm and coconut oil. Thus, it is safe to say that during this period, interesterification has become an important alternative method for producing structured fats instead of the process of partial hydrogenation because this modification does not lead to forming harmful trans isomers of fatty acids [41].

The enzymatic interesterification has several advantages for lipase’s high reaction efficiency, flexible reaction, and aforementioned fewer by-products [47]. In comparison with another modification, e.g., the chemical interesterification method (a randomised rearrangement process), using specific lipase during enzymatic interesterification allows better specificity over the reaction [48]. Immobilised enzymes are commonly used for enzymatic modification as immobilisation substantially increases the lipase’s stability at elevated temperatures in microaqueous systems as opposed to free form ones [49]. Lipases can be recovered through the physical process of filtration and can thus be reused [50]. According to the same authors, lipases such as sn-1,3 can direct the process and thus produce lipids with distinctive properties. Hence, enzymatic interesterification has found applications in the fat industry for producing specialty functional fats with specific TAG species. Significantly, the natural function of lipases is to catalyse fat hydrolysis reactions. Lipases act on the surface of the oil/water phase separation and must undergo certain conformational changes before the substrate can bind to the enzyme-active site mentioned earlier. Therefore, the water content is critical here. Limiting it in the system results in the dominance of the interesterification reaction over the hydrolysis reaction [51,52,53]. A certain minimum amount of water is necessary for the correct and efficient operation of the enzyme. Water in a reaction system serves two functions: first, it keeps the catalyst active and gives the enzyme molecule conformational flexibility; second, water is the reactant that releases fatty acids and incomplete acylglycerols (MAG, DAG) from triacylglycerols in the hydrolysis reaction.

Another important aspect of this process is that the biologically active FAs in the raw fat remain intact during fat modification. Therefore, enzymatic interesterification can be an opportunity to expand the use of waste animal fats, including sheep tallow, lard, and beef tallow [42,54]. In general, it is indicated that enzymatic interesterification makes it possible to obtain a new fat with new characteristics and improved physicochemical properties (i.e., slip melting point, polymorphism, texture, structure, consistency, and better oxidative properties) [55]. The above details are important not only for the use of fats as raw materials in the food industry, but evaluating their structure (e.g., the lengths of acid chains in TAG molecules) also determines their physical behaviour as dietary fats, as well as their behaviour during lipid digestion and other biological processes [56]. It is assumed that medium/long-chain TAGs can cause a significant reduction in fat accumulation [57].

Nowadays, enzymatic interesterification is commercially used to produce a variety of modified lipids, such as zero-trans and shortening-like margarine, cocoa butter substitutes and equivalents, low-calorie structural lipids, human milk fat substitutes, and edible films and coatings. Enzymatic interestrification has also found its way into proposed lipids with specific nutritional value and health effects. The growing interest in the nutritional importance of essential fatty acids such as medium chain FAs, caproic, caprylic, and capric acids, polyenic FAs (e.g., eicosapentaenoic (EPA, C20:5n-3), docosahexaenoic (DHA, C22:6n-3), linolenic (C18:3n-3) and *γ*-linolenic (GLA, C18:3n-6) acid has led to the synthesis of structured lipids with nutraceutical properties through their incorporation in TAG molecules [41].Furthermore, enzymatic interesterification, like chemical interesterification, can be utilised to produce special fat preparations for patients with digestive disorders to food containing fatty acids (MCFA). These acids are easily and quickly absorbed in the body, relieving the work of internal organs (pancreas, liver) that metabolise food. Thus, they are used as a quick source of essential energy. These metabolic properties of fatty acids have been used to obtain low-calorie, structured triacylglycerols [58].

The first to enter the global market for enzymatically interesterified fats using immobilised lipases were Unilever and Fuji Oil, companies that produced this type of fat on an industrial scale in the mid-1980s. Both companies used sn-1,3-regioselective lipase from *Rhizopus niveus* for this purpose, yielding substitute cocoa butter [59]. Bunge Loders Croklaan manufactures BetapolTM, an enzymatically interesterified fat-based product that has been on the market since 1995 [60]. Furthermore, enzymatic interesterification can be carried out to obtain human milk fat substitutes (Unilever’s BETAPOL). Saturated fatty acids, mainly C16 in the triacylglycerols of human milk fat, are in the sn-2 position, while in vegetable oil, these acids are mainly located in the outer positions. Therefore, it is possible to produce a human milk fat substitute from vegetable oils by employing a lipase specific to the sn-1.3 position of the triacylglycerol.

An example is the acidolysis of tripalmitate with unsaturated fatty acids [61]. A Danish-Swedish company carries the AAK name and, together with Enzymotec, forms the Advanced Lipids joint venture. Their product is InFatTM, a product used in the producing modified milk for infants and toddlers that has been on the market for more than 10 years. Another product from the same company, Bunge Loders Croklaan, is CoberineTM, a cocoa-butter substitute obtained by the enzymatic esterification of palm oil with stearic acid [62]. Products launched in 2002 by Archer Daniels Midland (ADM) were zero trans isomer margarines and shortenings containing enzymatically interestrified fat [63]. For this purpose, ADM used immobilised lipases produced by Novozymes, as Karlshamns AB had done a year earlier [64]. ADM currently manufactures a product line called NovaLipidTM, which has been in Europe since 2008. This line includes products based on enzymatic interestrified soybean, palm, and cottonseed oils. In 2002, Karlshamns AB was the first company in the world to launch a commercial production line using immobilised lipase produced by Novozymes [64]. One of the key challenges for the enzymatic interesterification process was fat production without trans isomers. The target shortenings without trans isomers are produced by Bunge Oils [62].

Recent years have seen a visible shift in the direction of manufacturing new fat products, with a clear emphasis on the use of enzymatic esterification, both in basic research and implementation [65]. Some products are already on the market, and others are being developed, with ongoing development of completely new products, taking into account current nutritional trends and their quality and safety. This is especially true of products free of trans isomers or shortening agents produced by enzymatic transesterification. The opening of the market to the consumer also makes it possible to modify a fat product’s functionality by improving its structure, texture, or triacylglycerol composition and to modify it by introducing additives into the final product, i.e., vitamins or plant sterols, thus increasing its nutritional value [66].

Despite the environmental and public health benefits, enzymatic modification still has limitations concerning the high cost of the enzymes and their poor stability. Furthermore, the disadvantage of enzymatic interesterification is the risk of cross-contamination among individual production batches due to the frequent use of reactors operating in a continuous system [67].

### 3.2. Oleogelation

Oleogelation is a novel method of solidifying liquid oils by immobilising the liquid TAGs within the network of the so-called oleogelator. This method is currently of interest among scientists because it offers the possibility of producing solid and semisolid lipid systems rich in unsaturated fatty acids; thus, it is a promising healthier alternative to conventional solid fats rich in SFAs and TFAs. Oleogels are viscoelastic systems of liquid oils structured as oleogelators. They have some functionalities like those of solid fats [4,68,69,70,71]. The biggest advantage of oleogelation is to keep the nutritional value of liquid oil without changes in the FAs composition of fatty acids, unlike hydrogenation and interesterification. The oleogelation is possibly due to the use of oil-soluble, self-assembled substances. The gel structure is thermoreversible. A 3-dimensional network created by oleogelators immobilises oil molecules. The phenomenon of entrapping oil in an oleogel system is based on various interactions such as van-der Waals, electrostatic hydrogen bonding, and π-π stacking [70,72,73,74]. Oleogels can be made even from relatively small additions of 1% structurant. The properties and consistency of oleogels can be designed according to need by selecting a specific oleogelator and its amount, method, and conditions of oleogel preparation and type of oil [4,75].

#### 3.2.1. Oleogelators

Oleogelator molecules create a structure or network in oil, which is a continuous phase of oleogel [64]. The most important criteria for oleogelators in the food industry are their safety and GRAS status. Moreover, the process should be lipophilic, thermoreversible, natural by origin, able to keep a stable structure of oleogel at the temperature of food storage, and efficient, structuring oil in the smallest amount possible [76]. Scientists divide oleogelators into two main groups: low molecular weight and high molecular weight.

Low molecular weight oleogelators most used in scientific research are plant and animal waxes, monoglycerides, fatty acids, fatty alcohols, *β*-sitosterols, *γ*-oryzanol and lecithin. For high molecular weight oleogelators, there are cellulose derivatives (ethylcellulose, hydroxypropylmethylcellulose, and methylcellulose) and proteins. Low molecular weight oleogelators are usually soluble by dispersion in heated oil. The temperature depends on the melting temperature of the oleogelator. With high molecular weight oleogelators, only ethyl cellulose oleogels can be developed by dispersing this compound in heated oil. The rest usually require an oil-in-water emulsion preparation and dehydration by drying or a solvent exchange method [73,76].

The choice of oleogelators is quite large, which helps to design better product properties. Each of them may create gels with different features and different ways of preparation [70,73,76].

##### Monoglycerides

Monoglyceride molecules consist of glycerol and one fatty acid. This substance is non-ionic and has hydrophobic and hydrophilic parts. MAG is a food additive used as an emulsifier in spreads, shortenings, milk, and bakery products. In the European Union, this additive has the symbol E471. One of the biggest advantages is that monoglycerides are widely available at a relatively low price. This compound may also occur naturally in oils, fats, and food products due to the hydrolysis of triacylglycerols. MAG can be diluted in heated oil at approximately 65 °C. While cooling down, the molecules form crystals, and on optical light micrographs, they can be observed as “needle-like” structures. According to most research, the critical concentration of MAG as an oleogelator in most oils is 3%. The properties of this oleogelator may change with its origin, which results in its composition [77,78]. The most popular MAGs available on the market are from palm oil, which is rich in palmic and oleic acid, or hydrogenated stearic acid. In the study of Zhang et al. [78], it was demonstrated that monooleic glycerides have weak crystallisation behaviour and do not form semisolid oleogel systems even at high concentrations of 15%. The same research demonstrated that saturated fatty acid glycerides formed oleogels at a concentration of 3%. However, it was shown that monocapryli and monostearate glycerides formed smaller crystals than monolauric glyceride, which is more desirable in food products.

##### Waxes

Natural waxes have plant and animal origins. Their chemical composition may consist mainly of esters of fatty alcohols and fatty acids, but they may also contain hydrocarbons, fatty alcohols, resins, ketones, and sterol esters. Their mechanism of oleogelation is similar to that of MAGs. They form a crystalline 3-D network, which entraps oil by absorbing it into pores [79]. In optical light micrographs, most of the waxes are seen in the form of “needle-like” structures. However, there can be some differences, which depend on their origin, purity, and concentration. In the micrographs presented in the research of Onacik-Gür et al. [68], it was seen that bleached white beeswax showed a denser network and shorter crystals than yellow beeswax. Candelilla wax has characteristic small “grain-like” crystals, and carnauba wax has spherical crystals. Other research revealed that with increased concentrations of wax crystals, the size of the crystals decreases, and the network becomes more ordered [70]. Waxes can form a semisolid oleogel structure at concentrations of 1–4%. Oleogels made of waxes forming long crystals have a less shiny surface. One of the most important features of oleogelators is their oil-binding capacity (OBC%). Waxes have better properties for holding oil in their structure than MAG [80,81]. In the study of Hwang and Winkler-Moser [82], it was found that mixtures of waxes may increase the firmness and melting point in comparison with single ones. Waxes that have GRAS status are beeswax, candelilla wax, carnauba wax, and rice bran wax [76]. The most common way to prepare wax-based oleogels is dispersion, similar to MAGs. Oils are heated to the melting temperature of the oleogelator, mixed, and cooled down [80,81].

##### Phytosterols

Phytosterols are natural substances in vegetable oils. They are added to functional products due to their cholesterol-lowering properties. The most studied sterols used as oleoegelators are *β*-sitosterol and *γ*-oryzanol. *β*-sitosterol occurs in most oils, while *γ*-oryzanol is typical for rice bran oil. They cannot form gels individually but only as a mixture or with an added surfactant. *β*-sitosterol and *γ*-oryzanol are self-assembling and create oleogels by co-crystallisation, developing a network of hollow tubes that immobilise oil by their capillary action [83,84].

##### Polysaccharides

The most described polysaccharide that forms oleogels is ethyl cellulose (EC), a cellulose derivative with a melting point of >130 °C [85,86].

Ethyl cellulose is a food additive used as an emulsifier. In the EU, it has the assigned number E462. EC can be used as an oleoegelator because it binds oil due to hydrogen bonds and van der Waals forces. The mechanical properties of EC oleogels depend on their molecular weight and the development of harder gels. The addition of surfactant modifies them by making the gels more elastic [85,86,87].

In recent years, researchers have developed new oleogels by employing hydroxypropyl methylcellulose (HPMC) and other cellulose derivatives. Unlike EC, they cannot be diluted in oil by heating and mixing. HPMC isused as an emulsifier, and then water is evaporated, or after hydrogel preparation, water is evaporated by lyophilisation, forming a sponge-like dry network filled with oil and homogenised [88,89].

##### Proteins

Proteins are not typical oleogels because they do not create a network in oil. Proteins are used as emulsifiers and surfactants in classical emulsions because of their amphiphilic nature. However, they can also be used as agents for developing a high internal phase Pickering emulsion. This feature lets us obtain oleogels by a method similar to the one for HPMC oleogels. In Pickering emulsions, oil droplets are surrounded on the surface by solid nanoparticles, microgels, or fibrils, which develop a barrier.

Moreover, this kind of emulsion can be characterised by enhanced stability because of the formation of a 3-dimensional viscoelastic particle network. Therefore, proteins that can be used in the Pickering emulsion development should have a tendency to aggregate [90,91]. One of the methods for developing a protein oleogel involves removing the water phase from the Pickering emulsion. The second way of preparing protein oleogels is the solvent exchange method. In the first stage of this method, a hydrogel is formed by the dispersion of proteins in water. To enable opening hydrophobic groups, the system is heated, and then a solvent with a medium polarity (acetone) is added stepwise to remove water. In the next step, the solvent is removed and replaced by oil. The final protein oleogel may contain <1% of water. The proteins that the researchers used were whey protein [92], soy protein isolate [93], *β*-lactoglobulin [94], and gelatin [95]. Protein oleogels are the least analysed but have great potential; however, their properties as fat replacers need to be studied.

#### 3.2.2. Effect of Type of Oil on the Properties of Oleogels 

Oil as a gelling solvent plays an important role in the development of oleogels’ properties. With wax oleogels, it was found that oils with a higher degree of saturation of fatty acids formed oleogels with increased strength [95]. This effect is reversed with EC oleogels. Oils rich in unsaturated FAs are more susceptible to oxidation, which increases their polarity. It was found that ethyl cellulose oleogels increase their strength in oils with higher polarity due to their better solubility [87]. The study of Zetzl et al. [86] found that pores in the structure of EC in oleogels decreased with a higher content of unsaturated FAs in oil. In the study of Valoppi et al. [96], it was found that with the increasing length of fatty acid chains, firmness and rheological parameters increased for MAG oleogels. The presence of polar components in oil increases the strength of sitosterol-oryzanol oleogels due to the expanded interactions [84]; these researchers also found that TAG composition has a minimal impact on phytosterol-oleogels. Similar results were found for wax olegels. With the increasing content of polar compounds in the formation of crystals, structures became highly ordered, and the critical concentration of wax to form a gel decreases [97]. However, the study observed that a lower content of polar compounds led to the development of the hardest beeswax oleogels and smaller crystals [75].

#### 3.2.3. Oleogels as an Alternative to Hydrogenated Fats

Oleogels are not yet used in the food industry. However, they are very promising solid fat substitutes. Oleogels may have similar technological properties as the fats used in the original product, but they are more nutritionally valuable than hydrogenated and solid fats. It was found that oleogels can improve the oxidation resistance of oil [98] and the lipid fraction in the product [99]. Publications show the successful replacement of solid fats in bakery products, spreads, sausages, ice cream, and chocolates (Table 2) [70,81,100].

In the study of Sun et al. [101], a functional dark chocolate was developed by substituting cocoa butter with 50% *β*-sitosterol-*γ*-oryzanol oleogel. The final product had similar texture, crystal structure, and sensory properties to 100% cocoa-butter dark chocolate. In the study of Li and Liu [102], it was found that monostearate corn oil oleogels at 100% to keep a solid form can replace cocoa butter. The advantage of replacing solid fats with oleogels is the reduction of saturated FAs and the delay of chocolate bloom, which is an important problem in this kind of product [103]. Finally, Alvarez et al. [104] reported that partial substitution of cocoa butter is possible by HPMC-sunflower oil oleogels. According to sensory analysis, a 70% replacement was possible; however, from a technological point of view, the replacement should not exceed 50% due to the weakening fat-crystal network.

Oleogels can furthermore be used as cream fillings in cookies and pralines. Monogyceride-high oleic sunflower oil oleogel was successfully used as a fat replacer in cookie cream and showed less migration than traditional creams.

Bakery fats and shortenings are well known for their high content of saturated fatty acids and trans fatty acids; hence the endeavour to develop healthier or vegan versions of common cookies and cakes. One of the ways is to replace solid fat with nutritionally valuable oils. Oleogels seem to be suitable for developing such products. According to the study of Mert and Demirkesen [105], partial replacement of shortening by carnauba wax-canola oil oleogels made no difference in the quality of cookies. Similar observations were reported for muffins with a 50% replacement by HPMC oleogel [106]. Some studies showed that 100% replacement of bakery fat in cookies was possible [17]. It was found that candelilla wax, ethyl cellulose, and monoglyceride oleogels are able to limit the migration of oil from biscuits [81].

The group of products using hydrogenated fat isexpanding. The study of Patel et al. [107] found it possible to formulate low fat spreads (35–42%) with shellac wax oleogels. Shellac stabilises oil as well as water droplets due to the presence of fatty alcohols. Researchers showed it possible to replace 100% partially hydrogenated palm oil in margarine with beeswax oleogel and hydrocolloid oleogel with no significant differences in the hardness of the product [108].

Other research shows the possibility of using oleogels as a frying medium. During frying, high temperatures of 150–190 °C are used. Under these conditions, oils rich in unsaturated fatty acids are oxidised much faster than hydrogenated fats and other solid fats. It was found that carnauba wax increased the smoke point of the frying medium [109]. Frying in oleogel has the further advantage of reducing of oil uptake [109,110,111]. In the study of Guneser et al. [112], it was found that beeswax oleogel, after 7 h of frying at 180 °C, had a lower content of polar compounds than sunflower oil. In the study of Lim et al. [110], instant fried noodles were stored and analysed; it was found that the peroxide value was lower in oleogels than in soybean oil but significantly higher than in palm oil.

**Table 2:** Potential possibility of solid fat replacement (including hydrogenated fat in similar commercial products) by oleogels.

Products—The Source of Saturated Fatty Acids and a Potential Source of Hydrogenated Fat (in Commercial Products)		Optimal Replacement of Solid Fat by Oleogels	Type of Oleogels (Oleogelator and Type of Oil)	References
Shortenings / bakery fats in bakery products	Cookies	40%	3% candelilla wax in canola oil and 70% replacement of shortening by 6% candelilla wax in canola oil	Mert&Demirkesen [105]
		100%	10% carnauba wax–insect oil (Tenebrio molitor)	Kim&Oh [113]
		70%	6% rice bran wax- rice bran oil oleogel	Pang et al. [114]
		100%	3% candelilla wax—high oleic rapeseed oil oleogel and 5% monoglycerides—high oleic rapeseed oil oleogel	Onacik-Gur&Zbikowska, [99]
	Muffins	100%	monoglycerides -high oleic sunflower oil oleogel	Giacomozzi et al. [115]
		50%	1% HPMC—sunflower oil oleogel	Oh &Lee [106]
		100%	Rapseed oil oleogels structured by 5% of candelilla, sunflower, yellow and white beeswax	Kupiec et al. [100]
	Bread	100%	10% of monoglycerides or rice bran wax—high oleic soybean oil oleogel	Zhao et al. [116]
	Buns	100%	HPMC+ xanthan gum—olive oil or high olei sunflower oil oleogel	Bascuas et al. [117]
	French pastry	50%	HPMC—high oleic sunflower oil oleogel	Espert et al. [118]
Chocolate spreads		50%	HPMC—olive/sunflower oil oleogel	Bascuas et al. [119]
		100%	20% Glycerol monostearate—corn oil oleogel (45% water in oleogel emultion)	Tirgarian et al. [120]
Margarines		100% replacement of partially hydrogenated palm fat	10% beeswax-sunflower oil oleogel hydrocolloid based oleogel (3.15% sodium caseinate, 0.5% guar gum, 0.22% xanthan gum)	Abdolmaleki et al. [108]
Creams and fillings		100%	monoglycerides–high oleic sunflower oil oleogel (100 g/kg)	Palla et al. [121]
Ice cream		50%	6% carnauba wax—soybean oil oleogel	Airoldi et al. [122]
		100%	7% beeswax—camellia oil oleogel	Jing et al. [123]
Frying medium		100%	3% an 8% beeswax—sunflower oil oleogel	Gunser et al. [112]
		100%	carnauba wax—canola oil (5 g/100 g and 10 g/100 g) oleogel	Adrah et al. [111]
		100%	carnauba wax—soybean oil (5 g/100 g and 10 g/100 g) oleogel	Lim et al. [110]

## 4. Conclusions

Solid fats are necessary to produce many products, including food. Therefore, they are important not only for practical use but also for nutritional value. The common utilisation of solid fats, especially in food, produced from oils by hydrogenation, interesterification, and fractionation processes is also of concern to consumers. These concerns are related to the high amounts of SFA in modified fats, the possible presence of TFA, and a small level of nutritionally valuable unsaturated FAs. Conventional methods of fat and oil modification, in particular partial hydrogenation, lead to a reduction in nutritional value. Therefore, different attempts are made to find alternative ways to produce solid fat with a low SFA content and without TFA.

Enzymatic interesterification is a modification that meets current environmental requirements and trends in product safety and sustainable production. The biologically active fatty acids in the raw materials remain intact after modification. One of the promising ways is to use oleogels. Thanks to small amounts of structure-forming substances, it is possible to obtain solid and semisolid fats with high nutritional value. In the future, oleogels may eliminate fats rich in saturated fatty acids and trans isomers from foods and reduce the need for palm oil. The possibility of designing the properties of oleogels by modifying the ingredients, preparation method, and conditions of the process, they can be utilised in different products. They can be helpful in the food industry (e.g., in bakery products, kinds of margarine, chocolates and chocolate-derived products, meat products) and other industries (e.g., cosmetics, pharmaceuticals). Therefore, it is advisable to disseminate modern fat modification techniques, such as enzymatic transesterification and the development of oleogelation. Fats modified by such methods may reduce the content of saturated FAs and TFAs in the diet and reduce the incidence of cardiovascular diseases.

## Figures and Tables

**Table 1:** Effect of process conditions and oil saturation on the hydrogenation process [3,25,27].

Increase of Parameter	H_2_ Concentration on the Catalyst (Ni)	*Cis/Trans* Isomerisation
Temperature	−	+
Mixing intensity	+	−
Catalyst concentration	−	+
Catalyst activity	−	+
Pressure	+	−
Degree of oil unsaturation	−	+

## Data Availability

Not applicable.
